# Anastrozole as add-on therapy for cabergoline-resistant prolactin-secreting pituitary adenomas: real-life experience in male patients

**DOI:** 10.1007/s11102-021-01165-0

**Published:** 2021-06-26

**Authors:** Filippo Ceccato, Laura Lizzul, Giacomo Voltan, Mattia Barbot, Carla Scaroni

**Affiliations:** 1 Endocrinology Department of Medicine DIMED, University of Padova, Padova, Italy; 2 Endocrine Disease Unit, European Reference Network On Rare Endocrine Conditions (endoERN) Center of Padova, University-Hospital of Padova, Padova, Italy; 3 Department of Neuroscience DNS, University of Padova, Padova, Italy

**Keywords:** Prolactin, Aggressive pituitary adenoma, Cabergoline, Anastrozole, Combined treatment

## Abstract

**Introduction:**

Prolactin-secreting adenoma (PRLoma) can present as large and invasive neoplasm, with increased markers of cellular proliferation. First-line approach is Dopamine Agonists (DAs) treatment; however, DA-resistance has been reported, especially in male patients. Estrogens induce lactotroph cell replication and PRL secretion: the use of anti-estrogen treatment in patients with PRLoma have been described in few cases. We reported our experience regarding treatment with the aromatase inhibitor anastrozole (ANA) as add-on therapy for male patients with DA resistant PRLoma.

**Materials and methods:**

We describe four male patients (26, 38, 29 and 19 years old at diagnosis), with PRLoma (median diameter 26 mm, PRL 7730 μg/L). They were resistant to cabergoline (CAB, > 2 mg/week) in terms of PRL secretion and tumor size reduction. ANA 1 mg/day was added to the maximum tolerated dose of CAB for at least 1 year. Magnetic Resonance was performed at baseline, after 6 months of CAB + ANA combination and every 12 months afterward.

**Results:**

PRL levels decreased in all patients after CAB + ANA (mean − 70%, range − 44/− 97%), achieving a normalization of PRL levels in one case. Tumor size decreased in all cases (mean − 47%, range − 24.5/− 68%). No severe adverse effects have been reported, a moderate weight gain has been observed in two cases.

**Conclusions:**

Addition of an aromatase inhibitor (ANA) to the dopamine agonist therapy improved the control of prolactin levels and induced tumour regression.

## Introduction

Prolactin (PRL)-secreting adenomas (PRLomas) are the most common pituitary adenomas [1]. Usually, they present a benign clinical course and are responsive to dopamine agonist (DA) therapy, especially cabergoline (CAB), in terms of PRL secretion, recovery of gonadal function and size-reduction [2], in particular if PRL levels are ≤ 1 μg/L shorlty after treatment [3]. Some patients often achieve sustained remission even after DAs withdrawal [4,5]. However, 10% of patients present DA-resistance [6,7], defined as a failure to normalize PRL levels or to achieve > 50% of tumor size shrinkage [2]. In men, PRLomas are almost ten times less frequent than in females and they are more frequently macro-adenomas [2], with higher PRL levels (> 1000 μg/L) [8], aggressive behaviour and DA-resistance [9,10]. Due to these aspects, in 2017 WHO classified PRLomas in men as a variant of aggressive pituitary adenomas [11]. In selected aggressive forms, resistant to high-dose DAs therapy, treatment is a challenge: the use of somatostatin analogs (octreotide [12] or pasireotide [13]), tyrosine kinase inhibitor (lapatinib, an oral epidermal growth factor receptor inhibitor [14]) or chemotherapy with alkylating agents (temozolomide [15]) has been recently proposed after surgery or radiotherapy failure.

Whether gender differences reflect a delayed diagnosis or different cellular pathogenesis is poorly understood. The PRL synthesis and secretion is controlled by estrogens, thyrotropin-releasing hormone, epidermal growth factor, and dopamine [2]. The interplay between PRL and sex hormones is complex: estrogens induce lactotroph cell replication through pituitary tumor transforming gene (PTTG); anti-estrogen treatment can revert it in ovariectomized rats, suggesting a role for selective anti-estrogen treatment in pituitary tumors [16,17]. A study by Munemura et al. showed that estrogens have a stimulatory effect on PRL secretion by uncoupling dopamine receptors from G-protein in rats [18]. In humans, similar findings were confirmed by the PRLoma growth during estrogen therapy in a transgender male-to-female patient [19]. Furthermore, testosterone could be aromatized to estradiol, giving male tumors an advantage on cell proliferation [20]. In men, a lower estrogen-receptor expression has been reported in aggressive PRLomas with DA-resistance [21].

To date, a few attempts with selective estrogen receptor modulators (SERMs) have been described, without promising results [22–24]. Aromatase inhibitors (ARIs) inhibit the transformation of testosterone to estradiol, and can have a role in lactotroph proliferation [25]. In 2002, a male patient with PRLoma treated with CAB showed a further PRL reduction with the association with the non-steroidal ARI anastrozole (ANA) [26]. Another case of ARI use (letrozole) was described more recently by Heidari et al., with comparable results [27].

Considering these isolated observations of combined therapy with ARIs and DAs playing a synergistic effect in males, we share our experience in 4 male patients treated with long-term CAB + ANA therapy.

## Patients, materials and methods

In our cohort of patients with PRLoma (94 females and 60 males, median follow-up 10 years, interquartile range: 6–14 years), four young males presented a macro/giant PRLoma, resistant to CAB medical therapy (> 2 mg/week); clinical details are reported in Table 1.

**Table 1:** Prolactin (PRL) levels and magnetic resonance (MR) size of pituitary tumors; Δ: percentual difference of PRLoma volume calculated between CAB alone (best diameter) and CAB + ANA treatment

Patient, age at PRLoma diagnosis	PRL (μg/L) baseline	MR baseline: Size (mm) – Volume (mm^3^)	Nadir of PRL level (μg/L) during CAB	Best MR size achieved with CAB: Size (mm) – Volume (mm^3^)	Last CAB dose prior to adding ANA (mg/week)	ANA treatment (months)	Nadir of PRL level (μg/L) during CAB + ANA; Δ reduction in brackets	*Best MR size achieved with CAB* + *ANA: Size (mm) – Volume (mm*^*3*^*); Δ volume reduction in brackets*	*Last CAB dose during CAB* + *ANA (mg/week)*
1, 26 years	14.000	33 × 23 × 35—14,133	1.920	20 × 19x29—5863	4.5	24	50 (Δ − 97.4%)	15 × 10x23—1835 (Δ − 68.7%)	3.5
2, 38 years	33.000	52 × 48x50—65,573	270	35 × 18x12—3972	4.5	56	23 (Δ − 91.5%)	30 × 17x10 —2681 (Δ − 32.5%)	4.5
3, 29 years	1.460	17 × 14x15—1735	35	13 × 8x10—506	3	30	18 (Δ − 48.6%)	11 × 6x6—193 (Δ − 61.9%)	2
4, 19 years	850	13 × 15x10—1091	27	13 × 5x11—400	3.5	15	14 (Δ − 44%)	12 × 5x9 —302 (Δ − 24.5%)	3.5

At baseline and during follow-up, serum PRL levels were collected with a stress-free sampling in the early morning through an indwelling venous catheter; the upper limit of normality (ULN) in males is 15 μg/L (mean of at least 2 measurements).

A contrast-enhanced pituitary magnetic resonance (MR) was performed at least three times: at baseline, after 6 months of CAB therapy at its higher dose and after at least 6 months of combined therapy with ANA.

Each MR underwent an operator-independent quantitative assessment by two authors (F.C. and L.L.) performed with Horos [28], a free medical image viewer, to calculate the volume of the mass.

A Goldmann perimetry of visual field was performed at diagnosis and repeated if clinically necessary. All patients were screened for valvular heart disease with transthoracic color Doppler echocardiography (at baseline, then every 24–36 months or in case of a new-onset heart murmur). The hepatic and renal functions were normal in all patients before the initiation of CAB and CAB + ANA. After the written acquisition of informed consent, all patients were treated with off-label ANA 1 mg/day in association with the maximum tolerated DAs dosage.

The cardio-metabolic profile was studied by systolic and diastolic blood pressure (SBP and DBP), heart rate (HR) and anthropometric measures at each visit, and biochemical blood sampling for fasting blood glucose, glycated hemoglobin A1c (HbA1c), lipid profile at baseline, after at least 6 months of CAB at its higher dose and after CAB + ANA association. Wearing light clothing and no shoes, participants were weighed and measured using a balanced beam scale and a vertical ruler. Weight was recorded to the nearest 0.5 kg and height to the nearest 0.5 cm. In accordance with WHO guidelines, waist circumference was measured at the end of natural breaths at the midpoint between the top of the iliac crest and the lower margin of the last palpable rib. We considered hypertension if SBP levels were ≥ 130 mmHg or DBP ≥ 85 mmHg (or if patients were receiving antihypertensive treatment). They were considered dyslipidemic if cholesterol or triglycerides were above normality (or being treated for dyslipidemia). The participants were considered diabetic if fasting plasma glucose was ≥ 5.5 mmol/L (or they were taking anti-diabetic drugs). BMD at lumbar spine (L1–L4) and femur (neck and total) was determined by DXA, using Hologic QDR 4500 C densitometer (Hologic Inc., Waltham, MA, USA).

Clinical data were collected in the web-based database of the University-Hospital of Padova, used as an electronic Case Report/Record Form (eCRF). The Ethics Committee of Padova University-Hospital approved the study.

## Results

### Patient 1

**Fig. 1:**
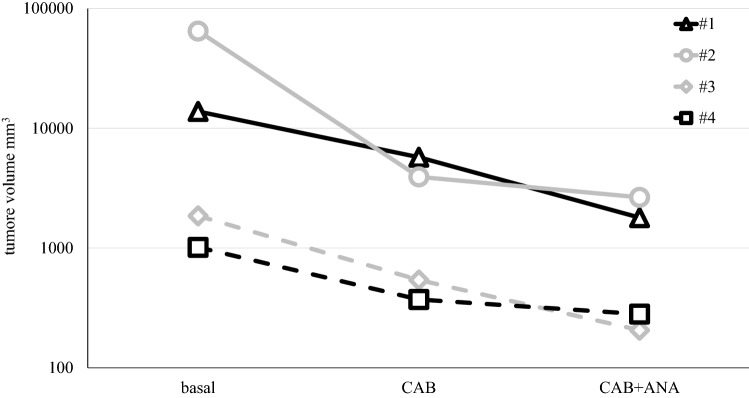
Tumor volume at baseline, after Cabergoline (CAB) and after combined Cabergoline + Anastrozole (CAB + ANA) treatment. Patient’s number is the same reported in Table 1. Tumor volume is depicted in logharitmic base 10 scale

A 26-year-old man complained of palpebral ptosis, headache, and visual impairment with bitemporal hemianopsia. A pituitary MR revealed a large (33 × 41x36mm) PRLoma with left cavernous sinus invasion. After 12 months of CAB treatment (up to 4.5 mg/week), PRL levels were reduced, without a significant shrinkage of the tumor. The patient then underwent to microscope-assisted trans-nasal surgery (TNS). The histological finding was consistent with an atypical adenoma (according to the WHO classification in use) [29]: positive PRL staining in more than 80% of cells, increased mitotic figures, MIB-1 > 3%, and positive p53. PRL levels did not improve after neurosurgery. After six years, he referred to our Endocrinology Unit, revealing persistently increased PRL levels (2245 μg/L) despite CAB treatment (4.5 mg/week), with the persistence of a large residual adenoma. Central hypothyroidism and hypogonadotropic hypogonadism were adequately substituted with oral L-thyroxine and transdermal testosterone (see Table 2). PRL levels before and after testosterone treatment did not change. Genetic evaluation of menin and AIP gene resulted wild-type, and an oral glucose tolerance test excluded a concomitant GH secretion. High dose CAB therapy was poorly tolerated due to orthostatic hypotension; echocardiography excluded valvular defects. We associated ANA to a reduced dose of CAB (3.5 mg/week): the patient achieved a PRL leverel reduction (145 μg/L) in 3 months, with a nadir of 50 μg/L, and a consistent shrinkage in tumor volume (see Fig. 1). After ANA initiation testosterone replacement therapy was maintained at the same dosage. ANA was well tolerated; a moderate increase of body weight was observed with combination treatment (detailed in Table 2).

**Table 2:** clinical parameters and endocrine evaluation according to treatment. CAB: cabergoline; ANA: anastrozole, ULN: upper limit of normality; RI: reference interval

Patient, age diagnosis	1, 26 years			2, 38 years			3, 29 years			4, 19 years		
	Baseline	CAB	CAB + ANA	Baseline	CAB	CAB + ANA	Baseline	CAB	CAB + ANA	Baseline	CAB	CAB + ANA
Weight (kg)	91	90	104	90	90	89	79	70	72	96	93	101
BMI (kg/m2)	25.2	25	28.8	35	35	34.7	27	23	24	26.1	26	27.4
Waist (cm)	100	103	110	100	103	104	102	96	94	102	102	107
Systolic/diastolic blood pressure (mmHg)	115/80	110/90	120/80	130/80	120/80	120/80	120/80	115/80	120/85	110/80	110/90	100/80
Fasting blood Glucose (mmol/L, RI 3.7–5.6)	4.6	4.5	4.5	4.5	4.1	4.2	4.6	4.4	5	4.7	4.2	4.7
HbA1c (mmol/mol, RI 20–42)	33	36	37	36	34	34	37	38	37	37	33	37
Cortisol (nmol/L, RI 138–624)	350	344	406	55	*Substitutive treatment*		328	342	415	302	272	358
Testosterone (nmol/L, RI 9.7–38.2)	*3.3*	*Substitutive treatment*		*4*	*Substitutive treatment*		2.35	15.5	20.3	13	21.99	13.42
fT4 (pmol/L, RI 9–22)	*8.8*	*Substitutive treatment*		*9*	*Substitutive treatment*		14.1	15	16	9.2	14	17.8
IGF1 ULN	1	0.8	0.6	0.4	0.6	0.5	1.1	1.2	1	1.1	1.6	1

### Patient 2

A 38-year-old man complained of headache with diplopia and oculomotor nerve palsy; at Emergency the CT reported a giant invasive sellar mass (60 × 60x72mm, with complete bilateral encasement of the cavernous sinus, showing lateral growth to the middle fossa and petrosal sinus). After surgical decompression (craniotomy approach), the patient developed anterior and posterior hypopituitarism; PRL levels before and after testosterone treatment did not show a significant change. Baseline PRL levels were extremely high (55,440 μg/L) and dropped only partially after surgery (33,000 μg/L), with a large residual adenoma (52 × 48x50mm). The patient received CAB (up to 5 mg/week), with partial control of PRL levels (270 μg/L).

A 12-months combined treatment with octreotide long-acting release (LAR, 30 mg monthly), achieved a nadir in PRL level (100 μg/L) and a partial response in tumor size (30 × 18x12mm). Somatostatin analog was discontinued for a progressive increase of liver enzymes, measured every 4 weeks: alanine transaminase 48 139 178 74 44 U/L (range 10–50), aspartate transaminase 30 67 80 42 33 U/L (range 10–45), γ-glutamyltransferase 47 170 158 101 61 U/L (range 3–65). This adverse event (grade 2 in the Common Terminology Criteria for Adverse Events Version 5.0) was asymptomatic, an abdominal ultrasound excluded gallbladder sludge and stones, and recovered after octreotide discontinuation. The rebound of PRL levels (930 μg/L) pushed us to start the association with CAB (4.5 mg/week) + ANA, resulting in a nadir of PRL levels of 23 μg/L and in a further size reduction of the pituitary adenoma.

Subsequent attempts to reduce the CAB dose led to a PRL escape, with good tolerability profile and absence of valvular defects. After ANA initiation the replacement therapy with 50 mg/day of transdermal testosterone was maintained. ANA was well tolerated; a progressive rise in bone turnover markers was observed, a dorsal spine radiography excluded vertebral fractures.

### Patient 3

A 29-year-old man suffering from migraine performed a cerebral MR with the incidental detection of pituitary adenoma (17 × 14x15mm, with bilateral cavernous sinus invasion and PRL 1460 μg/L); he also reported decreased libido and erectile dysfunction for two years. The genetic evaluation of AIP and menin gene mutations indicated wild-type genotype, an oral glucose tolerance test excluded an autonomous GH secretion; a hypogonadotropic hypogonadism was observed. A significant reduction of PRL levels (up to 35 μg/L) was achieved with CAB 3 mg/week, with the restoration of the normal gonadal function, without a consistent shrinkage of the pituitary lesion and with a poor dose tolerance due to hypotension and paroxysmal palpitations. The color-doppler echocardiography excluded valvular heart disease. The patient refused TNS. Therefore, to reduce drug-related side-effects, we started CAB + ANA with step reductions in CAB dose to 2 mg/week (-0.5 mg after 6 and 12 months), achieving a further reduction of PRL nadir to 18 μg/L, a shrinkage of the pituitary adenoma (up to 11 × 6x6mm) and a better tolerance of CAB.

### Patient 4

A 19-year-old boy, after a head trauma, performed MR with the incidental finding of a pituitary adenoma (13 × 15x10mm, PRL 850 μg/L). The genetic evaluation of AIP and menin gene mutations indicated a wild-type genotype, an oral glucose tolerance test excluded an autonomous GH secretion, the remaining pituitary function was normal. We started CAB at 1 mg/week with a further progressive increase to 3.5 mg/week reaching only a sub-normalization of PRL secretion (27 μg/L) with an absent control of the pituitary adenoma size (13 × 5x11mm). The patient refused neurosurgery; therefore, ANA 1 mg/day was added, achieving the normalization of the PRL levels and a reduction of the pituitary adenoma within the first 6 months of association therapy. No side effects have been reported, except a slight increase in body weight.

## Discussion

The treatment of patients with DA-resistant PRLoma is troublesome. In general DA treatment is recommended as the mainstay therapy, achieving biochemical remission in up to 80% of patients [30]. Neurosurgery is suggested not only in aggressive or large tumors, but also in non-invasive adenomas, for its capacity to achieve a sustained remission [31]. However, complications after pituitary surgery (hypopituitarism, infections, and cerebrospinal fluid leakage) can limit its indication [31], and in some cases, total removal of the adenoma cannot be performed, especially if the cavernous sinus is invaded.

Several definitions have been proposed to describe DAs resistance, one of the widely accepted is a failure to normalize PRL levels and to reduce tumor volume (at least 50%) with 2 mg/week of CAB [32]. In this setting, the most frequent clinical management is to offer TNS or to increase the dose of DA if tolerated.

The first report of a young male patient with DAs-resistant giant PRLoma treated with ANA was published in 2000. After the initial use of bromocriptine (with a consistent result on PRL secretion without adenoma shrinkage) CAB was initiated and increased up to 21 mg/week, achieving partial endocrine control. Since hypogonadism did not recover after PRL reduction, 3 attempts of testosterone substitutive treatment were done, observing a rise in PRL levels. Therefore, a selective ARI (ANA, 1 mg/day) was started and prolonged for 54 months achieving a decrease in PRL levels and a progressive reduction of CAB dose (to 5.5 mg/week) [26]. Afterwards, another case report was published, describing letrozole (2.5 mg/day) used as add-on therapy to bromocriptine and testosterone + hCG in a 36-years old man with a resistant PRLoma, with central hypogonadism and infertility. After 32 months of combination therapy, PRL decreased up to 75% and the sperm count improved leading to a spontaneous pregnancy [27]. This evidence pushed us to offer a combined approach with CAB and ANA in selected male patients with macro/giant PRLomas resistant to DA therapy, after unsuccessful or refused neurosurgery.

We report our experience with 4 patients, treated with combined CAB (maximum tolerated dose) + 1 mg ANA for at least one year. In our series, we observed a wide range of responses to CAB + ANA (from -44% to- 97% in terms of PRL secretion, mean -70%); one patient achieved a complete normalization of PRL levels. Regarding previously published case reports, a normalization of PRL levels was not reported [26,27]. Considering tumor volume, a mean shrinkage of 47% was observed (range of reduction from -25% to -69%). In patients with other pituitary adenomas (such as those GH-secreting [33]), at least ≥ 20% of tumor volume reduction is considered an effective endpoint. Given that the typical reduction of PRL secreting macro-adenoma with CAB is usually close to 60% in DA-sensitive forms [34], our experience shows promising results and could pave the way for a prospective study in DA-resistant patients. In our series we did not observe a complete resistance to CAB; nevertheless, in all cases, the highest tolerated doses of CAB (ranging from 3 to 5 mg/week) were able to achieve only a sub-normalization of PRL levels and a mild reduction of tumor burden. ANA association let a CAB dose reduction (1 mg/week) in two cases (patient #1 started with a lower dose, patient #2 reduced dose in the follow up) and increased adherence to treatment. Large and aggressive PRLomas present a male:female ratio of 9:1 [8], therefore our combined treatment could be considered at least in those adenomas with reduced likelihood of surgical remission.

It has been previously reported in the first case report that testosterone replacement therapy can promote a rise in PRL levels or adenoma growth (via the aromatization to estradiol) [26]. Patients #1 and #2 presented a central hypogonadism (secondary to surgery and elevated PRL levels); their testosterone levels were kept at the lower limit of the normal range with a transdermal dose sufficient to restore sexual function. Testosterone levels in patient #3 were normal after PRL reduction, as expected. Recently, it has been reported that ARIs can be used as an off-label treatment in patients with central hypogonadism, especially if testosterone could not be considered as replacement therapy (fertility preservation, prostate cancer, polycythemia, thrombophilia and severe cardiovascular disease) [35]. However, we did not observe a significant rise in androgen levels after ANA treatment, neither in eugonadal patients nor in the 2 patients with post-surgical hypogonadotropic hypogonadism (treated with transdermal testosterone). Replacement doses of testosterone were not modified during CAB + ANA therapy. Gonadotropin levels in the two eugonadal patients did not show significant changes after ANA.

In our series, high-dose CAB treatment (at least > 2 mg/week) was a selection criteria, therefore DAs-related side-effects are a matter of concern. Postural hypotension is not uncommon in ergot-based DA [36], and limited the use of high-doses CAB in 2 out of 4 patients. We did not observe any new-onset cardiac valvulopathy (an increased prevalence of tricuspid regurgitation has been reported with CAB, mediated by 5HT_2B_ receptors [37]) or impulsive control disorders and hypersexuality (that are more common in males, especially if current smokers or with high testosterone levels [38]). ANA was well tolerated, with no appearance of common acute side effects (such as nausea, headache, arthralgia, alteration of liver function markers) [39]. Pituitary function was assessed at baseline, during CAB treatment and after CAB + ANA association. We did not observe new-onset hypopituitarism or a modification in the substitutive treatment. In our series, we did not observe a change in SBP, DBP and HT after the ANA combination. In two patients (#1 and #4) we registered a sensible weight gain with the rise of body mass index (BMI) and enlargement of waist circumference after ANA. The fasting blood glucose and Hb1Ac levels maintained normal throughout the entire follow-up in all patients. The lipid profile did not show a consistent modification during the study. We study CAB + ANA association only in males; nonetheless, ARI are used also to treat estrogen receptor-positive breast cancer post-menopausal females, with a well-known safety profile: arthralgia, muscle pain and stiffness, reduction in lean body mass, mood swings, decrease in cognition and executive functioning, hot flashes, and night sweats [40]. Although aggressive PRLomas are more common in males, the combination of DAs and ARIs should be considered also in post-menopausal females.

Surgical management of PRLomas is effective in micro-adenomas or intra-sellar macroadenomas: remission-rate is elevated when the TNS procedure is performed by high-volume surgeons, and may be a cost-effective option in young patients [8,41]. In case of large or invasive adenomas, postoperative symptoms-mass or increased PRL levels arises from tumor remnants: high-dose CAB treatment is required, with increased risk of side-effects [37,42]. In our case-series, we described two different type of patients: #1 and #2 presented with with large and invasive PRLoma after surgical failure, requiring alternative therapeutic strategy for disease control and achieving a significant result (in terms of size and PRL secretion). On the other hand, patient #3 and #4 had smaller and less invasive lesions, and presented PRL levels close-to-normal with CAB. They both refused TNS, and CAB was not tolerated in one case. The therapeutic decision is a challenge in such cases, and in our opinion an effort to discuss these cases in a Multidisciplinary Team in a Pituitary Tumors Center of Excellence should be empowered [43].

Estrogens are essential for mineral metabolism and bone health in males. Estrogen determines the acceleration of bone elongation at puberty and epiphyseal closure, the achievement of skeletal proportions and peak bone mass, the maintenance of bone mass during adulthood and crosstalk with androgen for bone mass maintenance via the modulation of formation and resorption [44,45]. In our study, we do not observe a negative impact of ARI treatment on bone quality and metabolism. We observed in patient #2 a progressive rise of bone resorbtion markers since ANA was initiated, a dorsal spine X-ray excluded vertebral collapses and the DXA scan showed osteopenia. In this particular situation, we must also bear in mind the concomitant presence of hydrocortisone replacement therapy (20 mg/day) and the untreated GH deficiency as concomitant factors for bone damage. The remaining 3 patients did not show any impairment of bone metabolism: further studies with larger cohorts are needed to study the impact of long-term ARI treatment on fracture risk.

CAB can be used in monotherapy or associated with somatostatin analogs in acromegalic patients: it is a low-cost oral treatment, effective in selected cases; however, there is a paucity of evidence regarding its efficacy [46,47]. In our cohort, the addition of octreotide LAR to CAB resulted in a significant response in terms of PRL levels and adenoma size, as recently reported [12]; however, the development of liver function abnormalities resulted in somatostatin analog discontinuation. Estrogens antagonize GH receptor function, reducing hepatic IGF-1 synthesis: oral, but not transdermal, estrogen administration lowers circulating IGF-I [48]. The IGF-I suppression observed by the selective estrogen receptor modulators tamoxifen and raloxifen seems to correspond to those observed with oral estrogens: a randomized open-label study in 2018 reported that raloxifene could decrease serum IGF-1 level in acromegaly [49]. At the best of our knowledge, the use of ARI (alone or combined with CAB) is not reported in acromegaly, and should be considered in selected patients.

We are aware of the several limitations of this work. First, the observational and spontaneous design without a control group. Then, the small and heterogeneous cohort, with hypopituitarism acting as a confounding factors.

To conclude, we described an effective association-therapy with CAB and ANA. This option can lead to better control of PRL levels and to the shrinkage of tumor size, without serious adverse events. Its use should be considered for males or post-menopausal females with PRLomas, resistant to DAs, not a candidate or not willing a neurosurgical approach, especially when there is a high risk of iatrogenic hypopituitarism.

## Data Availability

All data generated or analyzed during this study are included in this article.
